# Impact of sedation, body position change and continuous positive airway pressure on distribution of ventilation in healthy foals

**DOI:** 10.3389/fvets.2022.1075791

**Published:** 2023-01-13

**Authors:** Muriel Sacks, Sharanne Raidal, Chee Sum Melanie Catanchin, Giselle Hosgood, Martina Mosing

**Affiliations:** ^1^School of Veterinary Medicine, College of Science, Health, Engineering and Education, Murdoch University, Perth, WA, Australia; ^2^School of Animal, Environmental and Veterinary Sciences, Charles Sturt University, Wagga Wagga, NSW, Australia

**Keywords:** CPAP, electrical impedance tomography, EIT, diazepam, recumbency, respiration, lung, spirometry

## Abstract

**Background:**

This study aimed to compare the distribution of ventilation measured by electrical impedance tomography (EIT), in foals under varying clinical conditions of sedation, postural changes, and continuous positive airway pressure (CPAP). To support the interpretation of EIT variables, specific spirometry data and F-shunt calculation were also assessed.

**Materials and methods:**

Six healthy Thoroughbred foals were recruited for this sequential experimental study. EIT and spirometry data was recorded: (1) before and after diazepam-sedation, (2) after moving from standing to right lateral recumbency, (3) in dorsal recumbency during no CPAP (CPAP_0_) and increasing levels of CPAP of 4, 7, and 10 cmH_2_O (CPAP_4_, _7_, _10_, respectively). Ventral to dorsal (COV_VD_) and right to left (COV_RL_) center of ventilation, silent spaces, tidal impedance variation, regional ventilation distribution variables and right to left lung ventilation ratio (R:L) were extracted. Minute ventilation was calculated from tidal volume (V_T_) and respiratory rate. F-Shunt was calculated from results of arterial blood gas analysis. Statistical analysis was performed using linear mixed effects models (significance determined at *p* < 0.05).

**Results:**

(1) Respiratory rate was lower after sedation (*p* = 0.0004). (2) In right lateral recumbency (compared to standing), the COV_VD_ (*p* = 0.0012), COV_RL_ (*p* = 0.0057), left centro-dorsal (*p* = 0.0071) and dorsal (*p* < 0.0001) regional ventilation were higher, while the right ventral (*p* = 0.0016) and dorsal (*p* = 0.0145) regional ventilation, and R:L (*p* = 0.0017) were lower. (3) Data of two foals for CPAP_10_ was excluded from statistical analysis due to prolonged apnea. Stepwise increase of CPAP led to increases of COV_VD_ (*p* = 0.0028) and V_T_ (*p* = 0.0011). A reduction of respiratory rate was detected with increasing CPAP levels (*p* < 0.0001).

**Conclusions:**

(1) In healthy foals, diazepam administration did not alter distribution of ventilation or minute ventilation, (2) lateral recumbency results in collapse of dependent areas of the lung, and (3) the use of CPAP in dorsal recumbency at increasing pressures improves ventilation in dependent regions, suggesting improvement of ventilation-perfusion mismatch.

## 1. Introduction

During the 1st year of life, the pulmonary capacity of foals' lags behind their body weight due to a dysanaptic growth of the lungs (1). This predisposes them to insufficient gas exchange leading to hypoxemia, hypercapnia and acidemia (2). These abnormalities can also be observed in foals with healthy lungs when undergoing clinical interventions such as sedation and postural changes (2).

Sedation with benzodiazepines is commonly used in healthy and diseased foals to enable short clinical interventions such as intravenous catheterization (3). Although the effects of intravenous diazepam on ventilation in foals is not reported, a foal's reduction in respiratory rate, as for midazolam, may interfere with their physiologically high minute ventilation (4). In adult horses, few cardiopulmonary changes are reported after intravenous diazepam administration (5). Intravenous diazepam at increasing doses from 0.05 to 0.4 mg/kg did not change respiratory rate but produced sedation, muscle fasciculations, ataxia and then recumbency at doses of 0.2 mg/kg and higher (6).

Furthermore, positioning sedated foals in lateral recumbency to facilitate diagnostic imaging, intravenous catheterization or intensive care procedures might lead to abnormalities in ventilation. Early respiratory studies in foals in 1984 reported arterial partial pressure of oxygen was, on average, 14 mmHg lower in samples taken in lateral recumbency compared to those taken 5 min after the foals stood up (7). Impaired gas exchange is documented in anesthetized horses when placed in dorsal recumbency, as evidenced by a rapid increase in alveolar-to-arterial oxygen tension difference (8, 9). This effect can be counteracted by the administration of positive pressure support ventilation (PPSV) such as continuous positive airway pressure (CPAP). CPAP is the delivery of continuous positive airway pressure during spontaneous ventilation, thereby reducing airway resistance and the work of breathing, which can improve oxygenation in dorsally recumbent adult horses (9). In foals CPAP was associated with increased oxygen extraction and more efficient carbon dioxide elimination (10).

The non-invasive real-time monitoring of ventilation by means of thoracic electrical impedance tomography (EIT) has been used in humans and mammals (11, 12). An electrode belt is placed around the thorax to detect ventilation dependent impedance changes across the pre-defined, species-specific lung field (*via* a finite element model), allowing for the distribution of ventilation to be assessed in real-time. In adult human patients, EIT has become popular for bed-side monitoring of mechanical ventilation, examining heart activity, lung perfusion and pulmonary function testing (11). In human neonates, EIT derived variables inform the clinician on regional lung volumes in diseased tissue during ventilatory support (11). Lung function and distribution of ventilation based on EIT have also been characterized in awake and sedated horses (12). There are no scientific reports published on the use of EIT in foals. Based on human and horse data, the stall-side use of EIT in foals might be a clinically useful diagnostic tool in the future. Novel insights of EIT variables in foals would provide better understanding of the impact of common clinical interventions on the pulmonary function of healthy foals. This would aid the clinician to guide sedation practice, choice of laterality and duration of recumbency and ventilatory support in the management of foals.

In this study, healthy foals were exposed to three different conditions: (1) before and after sedation with diazepam, (2) standing to lateral recumbency after additional sedation with intravenous fentanyl and xylazine, and (3) sequential increases in PPSV in sedated foals in dorsal recumbency. The aim of the study was to evaluate the effect of these conditions on the distribution of ventilation in foals using EIT, further supported by arterial blood gas analysis and spirometry methods.

## 2. Material and methods

The study protocol was approved by the Charles Sturt University Animal Care and Ethics Committee (ACEC A19218) and by the Murdoch University Research Ethics and Integrity Office (N3190/19). ARRIVE guidelines (13) were followed.

### 2.1. Animals

Six healthy, university-owned research foals (three fillies, three colts) with mean age of 25 days (range 9 to 30 days), median body weight 72.5 kg (range 68–95 kg) were included. All foals were of Connemara cross breeding (to Thoroughbred or Standardbred mares).

Foals were free of respiratory or systemic disease at the time of recruitment and for the duration of the study, based on thorough clinical examination, spirometry, and arterial blood gas analysis. All foals were part of a concomitant study, in which each foal underwent sedation twice in a randomized order and received two different modes of pressure support ventilation (14), with a 48 to 72 h wash out period between trials. For the present study, only data acquired during study arm one (CPAP) was used, and each foal served as its own control.

The six foals and their dams were maintained on pasture with supplementary lucerne hay to meet nutritional requirements and had free access to water at all times. Approximately 2 h prior to each study, they were brought into an individual mare and foal box. Except for time to collect initial data (described below), foals were allowed unrestricted nursing until immediately prior to sedation.

Each foal was manually restrained for physical examination and aseptic anaerobic collection of baseline arterial blood gas (ABG) samples from the carotid artery. A 14G, 89 mm catheter (Terumo Surflo, Macquarie Park, Australia) was aseptically placed in the jugular vein. The ideal position of the electrode belt was identified and marked by clipped hair (see below).

After preparation of the animals, the foal and its dam were brought to an outside area near the coumputed-tomography (CT) room, where the study held place. The mare was sedated intravenously (IV) with 200 mg of xylazine (Troy Laboratories Pty Ltd, Glendenning, NSW, Australia) and 10 mg of acepromazine (Ceva Animal Health, Pty Ltd., 11 Moores Road, Glenorie, NSW, Australia).

### 2.2. Study design

This longitudinal research study was performed with sequential conditions created by sedation, postural changes and ventilatory support.

#### 2.2.1. Part 1: Pre- and post-diazepam

**Figure 1:**
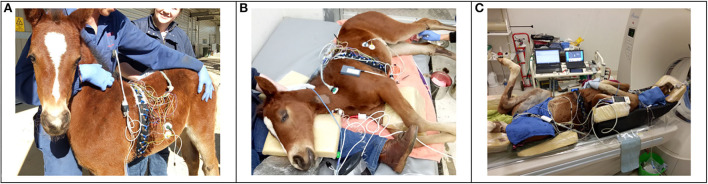
A foal undergoing study parts 1–3 is displayed. **(A)** Standing unsedated and with minimal restraint, with electrical impedance tomography (EIT) belt in place but not connected to the EIT software. **(B)** After a change from standing sedated to right lateral recumbency. To ensure an unchanged measurement plane, the EIT belt was repositioned to match the level of the clip marks in the coat. The face mask for spirometry measurements is not yet placed in this photograph. **(C)** In dorsal recumbency, with the EIT belt and a nasotracheal tube in place.

With the dam held in close proximity, the foals were instrumented with minimal restraint; EIT belt and face mask were applied and connected to the monitoring devices (EIT and spirometry). Several minutes were allowed for acclimatization. When the foals were breathing quietly, continuous measurements were performed over 2 min with the foals gently restrained with head and neck maintained in neutral (straight) position. Thereafter, the face mask was removed, and foals were sedated with diazepam (0.2 mg/kg; Parnell Laboratories, Alexandria, Australia) administered IV over approximately 30 seconds through the jugular vein catheter. The foals remained standing and were left undisturbed to sedate. Five min following drug administration, further measurements were recorded for 2 min. The EIT belt was left in place but disconnected from the EIT monitor, the face mask was removed (Figure 1). The dam was returned to the box.

#### 2.2.2. Part 2: Pre- and post-postural change

Immediately following recording after sedation with diazepam, fentanyl (0.005 mg/kg; Hospira, Melbourne, Australia) and xylazine (0.2 mg/kg, Troy Laboratories Pty Ltd, Glendenning, NSW, Australia) were administered IV. When ataxia was evident, the foals were manually placed in right lateral recumbency on a soft mat (Figure 1). After 10 min in right lateral position, the EIT belt was reconnected to the measurement device and the facemask was re-applied. Continuous measurements were recorded over 2 min. Arterial blood samples were collected aseptically and anaerobically from the left lateral metatarsal artery, blood withdrawal was started at the beginning of the 2 min recording time and was performed slowly over approximately 30 seconds.

#### 2.2.3. Part 3: Dorsal recumbency with 0, 4, 7, and 10 cmH_2_O CPAP

Immediately after data recording in right lateral recumbency, an intravenous continuous rate infusion of fentanyl (0.005 mg/kg/hr), xylazine (0.7 mg/kg/hr) and midazolam (0.1 mg/kg/h; Alphapharm Pty Ltd, Carole Park, Australia) in 0.9% sodium chloride delivered *via* syringe pump (Alaris IMED Gemini PC-1 infusion pump; VetQuip Pty Ltd., Erskine Park, NSW) was commenced and titrated to effect based on foal response including muscle tone, heart rate and respiratory rate, at a total flow rate of between 1.5 and 2 mL/kg/h for each foal. The trachea was intubated *via* the nasal passage with a naso-tracheal tube (NTT) inner diameter of 14 mm. Foals were placed on a CT table and positioned in dorsal recumbency. The cuff of the NTT was inflated once the foals were in stable dorsal position. A 22G, 2.5 cm polyurethane catheter (Terumo Surflo, Macquarie Park, Australia) was placed in the left or right facial artery to facilitate anaerobic arterial blood sampling.

Supplementary oxygen was administered at 5 to 10 L/min to achieve FiO_2_ of 50–60% during spontaneous respiration for 10 min, followed by 10 min of spontaneous respiration at FiO_2_ of 21% (room air). After these steps, resulting in a total of 20 min in dorsal recumbency, EIT and spirometry recording, blood gas sample collection and CT image acquisition were performed.

Respiratory support was provided using a portable multi-purpose ventilator (MTV1000 Dual Limb Ventilator System, MEK-ICS Company Ltd., Gyeonggi-do, Republic of Korea, distributed by Mediquip Pty Ltd., Loganholme, Queensland) with inspiratory and expiratory limbs and non-humidified room air. Inhalation and exhalation sensitivities for the flow trigger were left at default values (30%), and the apnea alert was set at 20 seconds. Apnea period lasting for more than 20 seconds initiated a switch to pressure support ventilation mode. In this case, foals were subsequently excluded from the study. Data of the time point of changed ventilation mode was excluded from statistical analysis. Stepwise increased levels of CPAP (CPAP at 4 cmH_2_O; CPAP_4_, CPAP at 7 cmH_2_O; CPAP_7_, CPAP at 10 cmH_2_O; CPAP_10_) were applied for 10 min each, after which EIT, spirometry and ABG sample collection was repeated as described above. The ventilation mode and the mean airway pressure was recorded for each measurement step for every foal. Data were averaged from ten artifact free spontaneous breaths.

Following recording of data at CPAP_10_, foals were returned to a mattress for recovery, the NTT and vascular catheters were removed. Foals were returned to their dams as soon as they were able to stand unsupported. A 24-h follow-up period was completed.

### 2.3. Data acquisition

#### 2.3.1. Electrical impedance tomography

##### 2.3.1.1. Verification of belt position

Prior to the study, EIT belt position was pre-defined based on thoracic radiograph studies of foals (MS), as described for dogs (15). The widest area between ventral diaphragm and cardiac silhouette was identified as the optimal transverse plane for evaluation of pulmonary tissue [or maximum lung:thorax area ratio]. Anatomic reference points to position the EIT belt at this level were identified in standing foals as the intersection between a horizontal line at the level of the tuber ischium and the 6th intercostal space. This point was marked on each side of the mid thorax by means of a clip mark in the coat. Low-conducting ultrasound gel was applied to the foal's hair circumferentially around the thorax to enhance electrode-skin contact. Thereafter, a custom-built EIT foal belt, equipped with 32 stainless steel washers serving as electrodes, was placed under slight tension around the thorax on top of the gel. The belt was made from stretchable elastic fabric such as commercially available as physiotherapy bands used for muscle strengthening exercises. An approximately 8 × 4 cm hard plastic buckle was used to close the belt over the withers. The belt was positioned in a vertical orientation through the two defined reference points. The cables connecting electrodes to hardware were wrapped in protective insulating synthetic fiber material and facing toward the tail of the foal.

##### 2.3.1.2. EIT data acquisition

The EIT belt was connected to the EIT device (BBvet, SenTec AG, Landquart, Switzerland) for single plane EIT data recording. When the foal was breathing quietly, EIT data were collected over 2 or 3 min or until at least ten motion artifact-free breaths were recorded. A modified Graz consensus reconstruction algorithm for EIT (GREIT) was used to generate 47 EIT images per second for each foal, representing breathing-related regional changes of impedance (16). Additional details on the use of EIT technology and image reconstruction are available (12).

**Figure 2:**
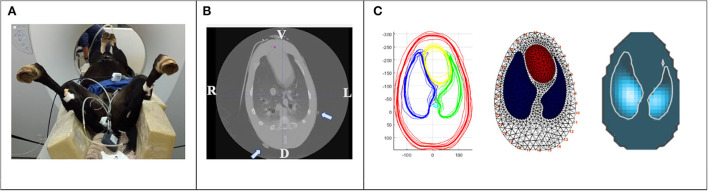
Creation of a foal specific finite element model. **(A)** Sedated foal in dorsal recumbency on a computed tomography (CT) table. A non-functional rubber belt with radio dense markers was placed around the thorax at the levels of the clip marks in the coat. Ventral lung regions are non-dependent, dorsal lung regions are dependent in relation to recumbency. **(B)** CT image of the thoracic cavity at the level of the non-functional rubber belt (radio dense markers are indicated by arrows). V; ventral, L; left, D; dorsal, R; right. **(C)** One CT slice of each foal at the vertical image plane of **(B)** were exported as DICOM files for segmentation. Average anatomic contours of the outer thorax (red), left and right lung (green and blue, respectively) and heart (yellow) were calculated (left), a finite element model created (middle) and EIT raw data reconstructed using this model (right).

Before commencing part three of the study, the EIT belt was removed, and the foal lifted onto the CT table in dorsal recumbency. To confirm optimal placement of the electrode belt, a non-functional rubber belt with mounted radio dense markers was placed around the chest at the level of the clip marks (Figure 2). Computed tomography images of the thorax were acquired in dorsal recumbency using a Toshiba Alexion 16-Slice Helical CT scanner with a slice thickness of 1.0 mm and slice interval of 0.8 mm (1.0 × 0.8). Respiratory movements were arrested by breath-hold induced by continuous pressure of the individual peak inspiratory pressure at each time point for the duration of image acquisition. The CT slice corresponding with the rubber belt position was used for the creation of the foal thorax finite element model (*see below*). Once CT data was acquired, the non-functional rubber belt was replaced by the EIT foal belt.

#### 2.3.2. Spirometry

For parts 1 and 2, spirometry was performed by placement of a tightly fitting veterinary anesthesia face mask (SurgiVet large canine mask, product number 32393B1; Sound Veterinary Equipment, Rowville, Vic) placed over the nostrils and muzzle to create an air-seal and to minimize dead space but enable normal abduction of the nares. The mask was attached to a combined spirometry and capnography connector which was connected to a human spirometer (NICO; Respironics, CT, USA). For part 3, the connector was directly attached to the NTT. Spirometry data were collected for each breath and recorded on a laptop using a dedicated software (Datacoll, Respironics, Wallingford, CT, USA). After each pressure change, the inspiratory and expiratory tidal volume were examined and the cuff of the endotracheal tube re-adjusted if the difference between inspiratory and expiratory volume was more than 10% of the tidal volume.

#### 2.3.3. Arterial blood gas analysis

Arterial blood gas samples were collected anaerobically from the carotid artery in standing foals, from the lateral metatarsal artery in foals in right lateral recumbency, and from the catheterized facial artery directly into heparinized syringes (BD heparinized syringes, BD, North Ryde, Australia) in dorsal recumbency. Catheters were flushed with heparinized saline after sample collection, and dead space fluid was discarded prior to sampling. All samples were analyzed (GEM Premier, Model 3500; Abacus ALS, Macquarie Park, Australia) within 5 min of collection, or stored on ice and analyzed within 60 min. Rectal temperature was measured and noted at each time point.

### 2.4. *Post-hoc* data analysis

#### 2.4.1. Electrical impedance tomography

##### 2.4.1.1. Creation of a finite element model

To enable the EIT software to reconstruct the impedance image with the foal specific anatomical lung regions, creation of a finite element model, or “mesh”, is required. In addition to improving image accuracy a finite element model allows the EIT software to recognize electrode position, which is particularly important in the case of poor electrode skin contact or failing electrodes (12, 17). A finite element thoracic model was constructed using the averaged information from the CT slice of each foal corresponding with the pre-defined ideal EIT belt position. The six CT slices were exported as DICOM files and heart, large thoracic vessels, lungs and thorax contours were segmented by ITK-SNAP (18). The segmented slice was exported as a vtk-file and Matlab software (Matlab, Mathworks, Natick, MA, USA) was then used to calculate an average contour from all six foals and the corresponding finite element model was created (Figure 2). All EIT raw data were reconstructed using this dedicated foal finite element model.

##### 2.4.1.2. EIT data analysis

Six to ten stable, sequential artifact-free breaths were retrospectively selected for each foal using dedicated software (ibeX, SenTec, Landquart, Switzerland). Of the selected breaths, conventional EIT variables were exported. Analyzed EIT variables included the ventral to dorsal (CoV_VD_) and right to left (CoV_RL_) centers of ventilation in percentage, dependent (DSS) and non-dependent silent spaces (NSS) in percentage and the right to left ratio of ventilation. Regional ventilation of the right and left lung was assessed in arbitrary units (AU) over four stacked regions in each lung [ΔZ(R or L)_V_, ΔZ(R or L)_CV_, ΔZ(R or L)_CD_, ΔZ(R or L)_D_] and tidal impedance variation (TIV) was recorded in AU as surrogate for tidal volume.

Briefly, the vertical (CoV_VD_) or horizontal (CoV_RL_) position of the CoV is expressed as a percentage of the ventro-dorsal or right-left extension of the lung region, respectively. Silent spaces represent the ventilation-induced impedance changes within the lung field below 10% of the maximum detected value. To describe the ventro-dorsal distribution of ventilation of the left and right lung during inspiration in more detail, the entire left and right lung fields were divided into four vertical portions of equal height [ventral 25% = ΔZ(Right or Left)_V_, centro-ventral 25% = ΔZ(R or L)_CV_, centro-dorsal 25% = ΔZ(R or L)_CD_, dorsal 25% = ΔZ(R or L)_D_]. Conventional EIT variables are explained in detail in previous reports (12, 19).

#### 2.4.2. Spirometry data analysis

Spirometry variables (expiratory tidal volume: V_T_ in mL and respiratory rate: RR in breaths per minute) were determined by post-sampling analysis over the measurement period using dedicated software (FlowTool Viewer 2.9.10, 2005; Respironics, CT, USA). Minute ventilation was calculated retrospectively for each time point.

#### 2.4.3. Arterial blood gas analysis

Analysis of temperature corrected arterial partial pressure of oxygen (PaO_2_), partial pressure of carbon dioxide (PaCO_2_), hemoglobin oxygen saturation (SaO_2_), hemoglobin (Hb) concentration allowed for retrospective calculation of the hemoglobin-based oxygen index F-shunt for standing unsedated and laterally recumbent foals (descriptive), as well as for all time points in dorsal recumbency under CPAP (statistical assessment).

The O_2_ content-based index (F-shunt) was calculated as: ([Cc′O_2_-CaO_2_]/[Cc′O_2_-CaO_2_] + 3.5 mL/dL) × 100; where 3.5 mL/dL is a fixed value of the difference between the arterial and mixed venous oxygen content (20).

Cc'O_2_ = Oxygen content of the pulmonary capillary blood, CaO_2_ = oxygen content of the arterial blood.

### 2.5. Statistics

All responses were considered continuous and followed a normal distribution based on visual inspection of Q-Q plots. Continuous, normally distributed variables are summarized as mean ± SD. Power analysis was performed to achieve power > 0.80 for the concomitant study investigating two different respiratory support mechanisms in foals (14). Based on results of previous studies of the same group, a sample size of six foals was able to detect differences in PaO_2_ and PaCO_2_ of 15 and 5 mmgHg, respectively, with a power > 0.80 and α = 0.05.

For part 1 and 2, linear mixed effects models using restricted maximum likelihood (REML) were used to evaluate a fixed effect of condition (pre-sedation, post-sedation, recumbency) on responses of interest. Foal was included as a random variance. Where there was a significant effect of condition, pairwise comparisons were made between conditions pre and post sedation, and between post-sedation (standing) and recumbency, against a Tukey-adjusted *p* < 0.05.

For part 3, linear mixed effects models using restricted maximum likelihood (REML) were used to evaluate a fixed effect of airway pressure on responses of interest. Foal was included as a random variance.. Where there was a significant effect of pressure, CPAP_4_, _7_ and _10_ were each compared against CPAP_0_, tested against a Dunnett-adjusted *p* < 0.05.

All analyses were performed using SAS 9.4 (SAS institute, Cary NC).

## 3. Results

All foals tolerated the interventions well, and no adverse effects were observed for the duration of the study.

### 3.1. Part 1: Ventilatory changes post-sedation

Distribution of ventilation, minute ventilation and tidal volume did not change significantly from pre- to post-diazepam sedation. Respiratory rate was lower in foals after diazepam sedation (*p* = 0.0004) (Table 1).

**Table 1:** Mean ± standard deviation for study parts 1 and 2; measuring electrical impedance tomography and spirometry variables and calculating F-shunt for six standing sedated foals and after 10 min in right lateral recumbency.

**Variable**	**Center of ventilation**		**Silent spaces**		**Regional ventilation**									**Spirometry**				

	**COV**_VD_	**COV**_RL_	**DSS**	**NSS**	Δ**Z*****R**_*V*_*	Δ**Z*****R**_*CV*_*	Δ**Z*****R**_*CD*_*	Δ**Z*****R**_*D*_*	Δ**Z*****L**_*V*_*	Δ**Z*****L**_*CV*_*	Δ**Z*****L**_*CD*_*	Δ**Z*****L**_*D*_*	**R:L**	**TIV**	**M**_V_	**V**_T_	**RR**	**F-shunt**
Pre-sed standing	60.29 ± 2.39	43.2 ± 4.8	3.84 ± 4.50	1.54 ± 1.91	7.53 ± 1.34	22.30 ± 6.69	23.69 ± 2.92	13.50 ± 6.31	0.54 ± 0.32	4.11 ± 2.63	17.98 ± 5.36	10.34 ± 2.21	2.21± 0.82	0.0055 ± 0.0015	25.86 ± 3.4	654.7 ± 123.6	42 ± 6.5^*^	10.44 ± 2.39
Post-sed standing	59.53 ± 0.94^£^	39.81 ± 3.91^£^	4.16 ± 5.58	1.55 ± 1.03	7.42 ± 1.68^£^	24.55 ± 3.24	26.91 ± 5.28	15.31 ± 3.81^£^	0.59 ± 0.61	3.29 ± 1.72	14.23 ± 5.01^£^	7.70 ± 2.13^£^	3.19± 1.23^£^	0.0075 ± 0.0016	23.12 ± 5.02	835.87 ± 217.5	27.85 ± 2.88^*^	NA
Post-sed lateral	64.38 ± 1.88^£^	53.69 ± 9.23^£^	8.45 ± 7.46	2.13 ± 2.84	4.26 ± 0.82^£^	16.18 ± 6.40	19.20 ± 9.06	6.01 ± 4.30^£^	0.33 ± 0.26	5.02 ± 3.22	29.24 ± 9.97^£^	19.76 ± 4.11^£^	0.95 ± 0.42^£^	0.0051 ± 0.0016	21.3 ± 4.02	648.30 ± 67.13	33.33 ± 3.69	16.12 ± 6.42

CoV_VD_, ventral to dorsal center of ventilation [AU]; CoV_RL_, right to left center of ventilation [AU]; DSS, dependent; and NSS, non-dependent silent spaces [%]; ΔZ(*R or L*)_*V*_, right or left ventral lung region [AU]; ΔZ(*R or L*)_*CV*_, right or left centro-ventral lung region [AU]; ΔZ(*R or L*)_*CD*_, right or left centro-dorsal lung region [AU]; ΔZ(*R or L*)_*D*_, right or left dorsal lung region [AU]; R:L, right to left ratio of ventilation; TIV, tidal impedance variation [AU]; M_v_, minute ventilation [L/min]; V_T_, tidal volume [mL]; RR, respiratory rate [breath per minute]; F-shunt [%], oxygenation index. Significant differences between pre- and post-sedation are indicated by ^*^ for study part 1; and between post-sedation standing versus post-sedation lateral recumbency by ^£^ for study part 2.

### 3.2. Part 2: Ventilatory changes post-recumbency-change

The CoV_VD_ (*p* = 0.0012) and CoV_RL_ (*p* = 0.0057) were higher after the foals were placed in right lateral recumbency, indicating a shift of ventilation toward the dorsal and left areas of the lungs. A decrease in ΔZR_V_ and ΔZR_D_ (*p* = 0.0016 and 0.0145, respectively) and an increase in ΔZL_CD_ and ΔZL_D_ (*p* = 0.0071 and *p* < 0.0001, respectively) were detected. Right to left ratio was lower in right lateral recumbency (*p* = 0.0017). Silent spaces and spirometry parameters did not change significantly (Table 2). F-shunt increased by 6% from standing unsedated to sedated foals in right lateral recumbency.

**Table 2:** Electrical impedance tomography and spirometry variables (mean ± standard) measured in six foals in dorsal recumbency without positive pressure support ventilation (CPAP_0_), and after 10 min of each continuous positive airway pressure at 4 cmH_2_O (CPAP_4_), 7 cmH_2_O (CPAP_7_), and 10 cmH_2_O (CPAP_10_).

**Variable**	**Center of ventilation**		**Silent spaces**		**Regional ventilation**									**Spirometry**				

	**COV**_VD_	**COV**_RL_	**DSS**	**NSS**	Δ**Z*****R**_*V*_*	Δ**Z*****R**_*CV*_*	Δ**Z*****R**_*CD*_*	Δ**Z*****R**_*D*_*	Δ**Z*****L**_*V*_*	Δ**Z*****L**_*CV*_*	Δ**Z*****L**_*CD*_*	Δ**Z*****L**_*D*_*	**R:L**	**TIV**	**M**_V_	**V**_T_	**RR**	**F-shunt**
CPAP_0_	57.07 ± 2.61^*, §^	41.16 ± 5.27	2.73 ± 3.00	3.68 ± 3.99	5.99 ± 3.13^*^	28.01 ± 8.28	27.76 ± 4.86	7.97 ± 6.45	5.05 ± 4.83	17.71 ± 4.98	6.74 ± 4.74	5.99 ± 3.13	2.75 ± 1.70	0.005 ± 0.0024	13.16 ± 2.15	564.79 ± 129.80^*, §^	29 ± 7^*, §, ♠^	14.65 ± 3.32
CPAP_4_	60.03 ± 1.11	37.99 ± 3.94	1.40 ± 1.69	4.06 ± 3.91	4.30 ± 2.15	23.32 ± 5.75	33.96 ±1.93	14.26 ±5.32	0.62 ±1.38	4.56 ± 4.33	14.23 ±4.10	4.75 ±2.23	3.65 ±1.61	0.006 ± 0.0023	11.01 ± 1.97	753.85 ± 92.24	15 ± 2^*^	14.83 ± 2.91
CPAP_7_	60.22 ± 2.34	41.39 ± 3.86	1.16 ± 0.91	5.78 ± 4.96	3.82 ± 0.89	24.65 ± 2.55	31.42 ± 6.14	10.03 ± 5.99	0.41 ± 0.96	3.95 ± 3.74	17.08 ± 4.03	8.63 ± 5.50	2.66 ± 1.20	0.007 ± 0.0022	10.59 ± 2.39	921.43 ± 145.20^*^	12 ± 3^§^	12.94 ± 2.46
CPAP_10_	62.90 ± 1.59^*^	43.21 ± 5.41	1.13 ± 1.37	8.67 ± 3.56	2.29 ± 0.53^*^	20.59 ± 4.93	31.83 ± 5.03	12.31 ± 4.41	0.53 ± 1.06	4.22 ± 4.32	18.19 ± 6.95	10.14 ± 3.43	3.14 ± 1.54	0.008 ± 0.002	10.76 ± 1.14	969.98 ± 221.34^§^	11 ± 2^♠^	11.08 ± 2.21

Additionally, F-shunt for each CPAP level is shown. Periods of apnea for more than 20 seconds triggered a switch to pressure support ventilation (PSV) in two foals at CPAP_10_, data sets of these two foals at CPAP_10_ were excluded from this table. CoV_VD_, ventral to dorsal center of ventilation [AU]; CoV_RL_, right to left center of ventilation [AU]; DSS, dependent; and NSS, non-dependent silent spaces [%]; ΔZ(*R or L*)_*V*_, right or left ventral lung region [AU]; ΔZ(*R or L*)_*CV*_, right or left centro-ventral lung region [AU]; ΔZ(*R or L*)_*CD*_, right or left centro-dorsal lung region [AU]; ΔZ(*R or L*)_*D*_, right or left dorsal lung region [AU]; R:L, right to left ratio of ventilation; TIV, tidal impedance variation [AU]; M_v_, minute ventilation [L/min]; V_T_, tidal volume [mL]; RR, respiratory rate [breath per minute]; F-shunt [%], oxygenation index. Significant differences between CPAP_0_ and CPAP_4_, CPAP_7_ and CPAP_10_ are indicated by ^*^, ^§^, ^♠^.

### 3.3. Part 3: Ventilatory changes in response to CPAP

All foals were breathing spontaneously on CPAP_4_ and CPAP_7_. At CPAP_10_, apnea of > 20 seconds was detected in two foals, which triggered the ventilator to switch to PSV. Data of these two foals for CPAP_10_ was excluded from the statistical analysis.

Stepwise increase of CPAP over the three pressure levels led to an overall increase of COV_VD_ (*p* = 0.0028), with a change observed between CPAP_0_ to CPAP_10_ (*p* = 0.0017). There was an increase in right ventral regional ventilation between CPAP_0_ and CPAP_10_ for ΔZR_*V*_ (*p* = 0.0351), with no other significant changes in regional ventilation variables. Tidal volume increased with increasing airway pressures (*p* = 0.0003), with increases from CPAP_0_ to CPAP_7_ (*p* = 0.0027) and from CPAP_0_ to CPAP_10_ (*p* = 0.0023). A reduction of respiratory rate was detected with increasing CPAP levels (*p* < 0.0001)_._ A lower respiratory rate was detected between CPAP_0_ and CPAP_4_ (*p* = 0.0002), CPAP_0_ and CPAP_7_ (*p* < 0.0001) and CPAP_0_ and CPAP_10_ (*p* < 0.0001)_._ No significant differences were detected in COV_RL_, DSS and NSS, right to left ratio, minute ventilation and F-shunt. The linear regression model did not reveal any association between F-shunt and any of the EIT variables.

## 4. Discussion

This study aimed to compare the distribution of ventilation measured by EIT, supported by spirometry data and F-Shunt in foals under varying clinical conditions of sedation, postural changes, and continuous positive airway pressure (CPAP). For a feasible application of EIT in foals at rest and during clinical interventions, several preconditions needed to be fulfilled: the creation of a CT-based foal specific finite element model, development of an EIT belt suitable for foals and confirming ideal EIT position around the thoracic cavity and relative to external landmarks were all achieved. Through measurement of EIT variables across various physiologic conditions imposed on the foals, (1) the administration of diazepam did not alter the distribution of ventilation or minute ventilation in healthy foals; (2) a change from standing to right lateral recumbency subsequent to additional sedation with fentanyl and xylazine led to a shift of ventilation toward the non-dependent lung; and (3) in dorsal recumbency increasing airway pressures during CPAP led to a shift of ventilation toward the dependent parts of the lungs. In two of six foals, CPAP of 10 cm H_2_0 was associated with prolonged apnea.

Using an EIT belt made of stretchable material with a fast release option is essential for the safety of foals and handlers. In this study, no evidence of impaired thoracic expansion was observed, which is especially important considering the high compliance of a foal's rib cage. Both the practical collection and off-line analysis of EIT data in healthy unsedated foals were possible with little modification of the commercially available equipment. The optimum belt position was predicted by radiographic chest studies of foals and exterior landmarks were defined. Those landmarks were confirmed by CT evaluation, identifying the transverse plane of greatest lung:thorax ratio averaged from the six foals. Observed impedance changes fitted within the regions of interest as defined by the newly created foal finite element model (Figure 2). One limitation was, that the EIT belt had to be taken off between measurement time points. This was addressed by clip marks in the hair coat, which allowed for correction of belt position and for accurate placement after temporary removal. Nonetheless, consistency of the measured anatomical lung area cannot be entirely controlled.

### 4.1. Diazepam sedation

Diazepam at the dose used in the present study is widely considered an appropriate choice of sedation in young foals (4, 21). While there is some scientific data available on the cardiopulmonary effects of intravenous diazepam in healthy adult horses, no direct scientific evaluation of the effects of diazepam on ventilation in foals has been published (5, 6). Our data confirmed anecdotal impressions and clinical observations that intravenous diazepam does not impact ventilatory efficacy in foals. While respiratory rate decreased, tidal volume increased thus maintaining minute ventilation relatively constant. Importantly, also the distribution of ventilation remained unchanged suggesting little alteration of gas exchange. However, arterial blood gas collection was not repeated in standing sedated foals precluding a direct comparison of blood gases between unsedated and sedated foals. Furthermore, the maximum age difference of 21 days between the youngest and oldest foal might indicate, that diazepam had variable cardiopulmonary effects dependent on the achieved level of sedation.

### 4.2. Right lateral recumbency

Ventilation shifted toward the left (non-dependent) and dorsal lung regions within 10 min of right lateral recumbency in all six foals. In the dependent lung, ventilation “centralized” around the centro-ventral and centro-dorsal lung regions, and lung collapse in this lung increased as shown by an upward trend in DSS. In anesthetized mechanically ventilated ponies in right lateral recumbency a shift toward the non-dependent lung was seen over the first 30 min without a change over the subsequent 30 min (22). In anesthetized rhinoceroses in lateral recumbency, similar shifts of ventilation toward the hilus in the dependent, and toward dorsal lung regions in the non-dependent lung were documented (23). The similarity of their gastrointestinal tract and diaphragmatic anatomy might be responsible for comparable changes in response to lateral recumbency in rhinoceroses and equids. As they belong to the taxonomic order of *Perissodactyla* the diaphragmatic flexure of the dorsal large colon lies within the intrathoracic part of the abdominal cavity in close contact with the diaphragm in standing animals (24). In laterally recumbent foals, the dependent highly compliant thoracic wall is limited in its movement by direct contact to the ground (25). In addition, the large colon might impede movement of the dependent diaphragm and thus further restrict lung expansion (23). Although not confirmed in a subsequent study, it was reported that foals younger than 7 days of age had an even higher tendency of lung collapse in lateral recumbency diagnosed on CT (26, 27). In our study the youngest foal was measured at 9 days of age and did not have greater lung collapse than older foals based on changes in silent spaces. Age-related differences in sedated foals have been attributed to suspected differences in chest wall compliance and specific lung compliance in younger foals (27, 28).

The rapid shift of ventilation after body position change was expected, based on previous reports in rhinoceroses as well as thoracic CT evaluation of ventilation in anesthetized healthy cats. In cats, the change in position led to higher attenuation and lower lung volume in the dependent immediately after positioning and remained static thereafter (29). The short time to notably reduced ventilation and lung collapse of the dependent lung in the present study indicates the importance of the positioning of recumbent foals in a clinical setting.

The variance in DSS prohibited the finding of a significant difference across conditions for this variable. The variance reflected inter-subject variability in the effects of right lateral recumbency on lung aeration and gas exchange in foals. This is confirmed by the wide range of F-shunt. In one foal, a small 2% DSS was associated with a minimal F-shunt of 10%, while in a foal with larger 8% DSS the highest individual F-shunt of 28% was detected. Importantly, the association between F-shunt and the increase in right to left ratio of ventilation in right lateral indicates that the change in distribution of ventilation had a direct negative impact on gas exchange under the conditions of this study. It remains uncertain, if the additional sedation in the form of intravenous fentanyl and xylazine administered prior to part 2 of the study influenced the distribution of ventilation once in lateral recumbency. This drug combination was chosen to achieve sufficient sedation for a prolonged time in dorsal recumbency during data collection. Additional studies specifically investigating the impact of these drugs on ventilation in healthy foals would be needed to minimize a confounding effect on our findings in the comparison of standing and right lateral recumbency.

In conclusion, the short time elapsed until notably reduced ventilation and lung collapse of the dependent lung indicates a potential deleterious effect of positioning sedated foals in lateral recumbency. This finding highlights the importance of deliberate timing of duration in lateral recumbency especially in diseased foals. Sternal recumbency enabling both lungs to be ventilated equally seems to be preferable but needs to be confirmed in future studies. Furthermore, our results endorse the usefulness of EIT as a non-invasive stall-side monitoring tool in foals, providing a breath-by-breath analysis of what otherwise can only be achieved by frequent arterial blood gas analysis.

### 4.3. Continuous positive airway pressure

Distribution of ventilation moved toward the dependent dorsal lung regions in foals in dorsal recumbency with increasing airway pressures as documented by increasing COV_VD_. This is in agreement with findings in anesthetized dorsally recumbent horses, where CPAP of 8 cmH_2_O resulted in more ventilation in dependent lung regions compared to horses without CPAP, causing an improvement of ventilation-perfusion matching in the CPAP group (30). As discussed earlier, rapid alveolar collapse in dependent lung regions was documented in ponies in dorsal recumbency when mechanically ventilated (22). It has been proposed that spontaneous breathing allows the diaphragm to move physiologically thereby allowing more expansion of dependent lung areas (31). The combination of the continuous airway pressure preventing collapse of small airways and the physiologic motion of the diaphragm during spontaneous breathing may be the reason for this beneficial shift of ventilation toward dependent lung regions. In a previous study comparing CPAP to nasal O_2_ insufflation in foals with pharmacologically induced respiratory suppression, CPAP but not O_2_ insufflation was able to increase arterial oxygenation, CO_2_ elimination and O_2_ extraction in foals (10), however only one level of airway pressure was examined in that study. In the current study, baseline measurements were taken in foals with no CPAP. Increasing airway pressure levels led to a successive increase of COV_VD_ and improvement of F-shunt, indicating a progressive increase of ventilation and gas exchange in more dependent lung areas such as described in pulmonary recruitment maneuvers (27). The recruitment of lung areas with increasing CPAP levels has not yet been described in horses or foals. A significant increase in right ventral regional ventilation between CPAP_0_ and CPAP_10_ was detected and was likely linked to a compensatory shift after a previous decrease in right lateral recumbency.

In foals in dorsal recumbency, CT revealed increased lung densities indicative of consolidation in the dependent portions of the lungs, relative to the nondependent portions of the lungs (26). These findings were considered most consistent with atelectasis, as previously described (26). A subsequent study by the same author demonstrated that alveolar recruitment maneuvers at increasing peak airway pressures to a maximum of 30 cmH_2_O led to marked increase in attenuation of the non-dependent lung regions, likely associated with hyperaeration due to over-distension (27). Although not significantly different between time points, DSS decreased and NSS increased in all six foals. EIT studies of greater scheme may discriminate relevant changes of silent spaces with increasing airway pressures and inform the clinician on the ideal airway pressures to improve dependent alveoli collapse while not over distending the non-dependent lung areas.

PPSV delivered to foals in dorsal recumbency in the form of CPAP was well tolerated by all foals at CPAP_4_ and CPAP_7_ but induced prolonged apnea in one third of the foals at CPAP_10_. A significant reduction in respiratory rates was previously shown for foals receiving CPAP at 10 cmH_2_O delivered by face mask (10). Apnea might be attributed to activation of the Hering-Breuer reflex in response to direct activation of airway stretch receptors due to tissue over-distension or increased mean airway pressure.

Although we were able to demonstrate application of EIT in foals and detect some changes across parameters measured under certain physiologic conditions, variability across a restricted sample size prohibits making definitive conclusions for some parameters such as NSS and DSS. The sequential order of standing to right lateral recumbency, and from right lateral to dorsal recumbency was not randomized due to the study design of the concomitant project. Previous EIT studies in adult horses demonstrated that the right lung generally contributes more than the left to ventilation in standing horses (19, 32). The six foals were placed in right lateral recumbency, consequently affecting ventilation in the larger lung. It remains unknown, to which degree the laterality of recumbency influences ventilation and gas exchange in healthy foals, and thus if one side should be favored over the other.

Although small, there was an age difference of 21 days between the youngest and oldest foal, potentially introducing age-related physiologic differences in ventilation. Future studies investigating the influence of aging lungs on the distribution of ventilation in foals are warranted.

In conclusion, EIT was deemed a useful and feasible monitoring tool for the investigation of distribution of ventilation in foals. Diazepam sedation had no influence on the distribution of ventilation and despite lower respiratory rates, minute ventilation did not change significantly in standing sedated foals. Right lateral recumbency of only 10 min duration led to a shift of distribution of ventilation toward the dorsal non-dependent lung regions, and to centralized and overall reduced ventilation in the dependent lung. In foals positioned in dorsal recumbency CPAP_10_ led to a shift of ventilation toward the dependent lung regions and improvement in gas exchange, however CPAP of 10 cmH_2_O induced prolonged apnea in two out of six foals. The outcome of this study confirms previous reports on minimal influence on ventilation of diazepam sedation in foals and underlines the rapid reduction of ventilation in the dependent lung regions in lateral recumbency. In dorsal recumbency, EIT detected a shift of ventilation toward the dependent areas of the lungs during CPAP, consistent with opening of the dependent areas of the lung which could lead to improvement of gas exchange. Further studies in lung diseased neonatal foals are warranted to evaluate the positive effect of CPAP.

## Data availability statement

The original contributions presented in the study are included in the article/supplementary material, further inquiries can be directed to the corresponding author.

## Ethics statement

The animal study was reviewed and approved by Charles Sturt University Animal Care and Ethics Committee (ACEC A19218) and by the Murdoch University Research Ethics and Integrity Office (N3190/19).

## Author contributions

MS, SR, CC, and MM collected the data. MS, SR, and MM analyzed data. GH performed statistical analysis. MS and MM drafted the manuscript. All authors interpreted results, contributed to conceptions of research and study design, and edited, revised, and approved the final version of the manuscript.
